# Supplementary material for: Multi‐dataset identification of innovative feature genes and molecular mechanisms in keratoconus
Source: J Cell Mol Med. 2024 Sep 19;28(18):e70079. doi: 10.1111/jcmm.70079 (PMC11412914; doi:10.1111/jcmm.70079)
Supplement: Supplementary file 2 — Material S2. [file JCMM-28-e70079-s002.zip › 7.GSEA analysis/4.MYRF/KEGG_ALANINE_ASPARTATE_AND_GLUTAMATE_METABOLISM.html]

Details for gene set KEGG\_ALANINE\_ASPARTATE\_AND\_GLUTAMATE\_METABOLISM[GSEA]

|  || Dataset | MYRF.group.cls#h\_versus\_l |
| Phenotype | group.cls#h\_versus\_l |
| Upregulated in class | h |
| GeneSet | KEGG\_ALANINE\_ASPARTATE\_AND\_GLUTAMATE\_METABOLISM |
| Enrichment Score (ES) | 0.23231883 |
| Normalized Enrichment Score (NES) | 0.7605773 |
| Nominal p-value | 0.81512606 |
| FDR q-value | 1.0 |
| FWER p-Value | 1.0 |
Table: GSEA Results Summary

  

Fig 1: Enrichment plot: KEGG\_ALANINE\_ASPARTATE\_AND\_GLUTAMATE\_METABOLISM      
 Profile of the Running ES Score & Positions of GeneSet Members on the Rank Ordered List

  

| SYMBOL | TITLE | RANK IN GENE LIST | RANK METRIC SCORE | RUNNING ES | CORE ENRICHMENT || 1 | DDO | na | 710 | 0.515 | 0.0973 | Yes |
| 2 | GPT | na | 789 | 0.498 | 0.2088 | Yes |
| 3 | AGXT2 | na | 3335 | 0.279 | 0.1992 | Yes |
| 4 | IL4I1 | na | 4670 | 0.233 | 0.2138 | Yes |
| 5 | GAD1 | na | 5643 | 0.204 | 0.2323 | Yes |
| 6 | GAD2 | na | 8016 | 0.138 | 0.1955 | No |
| 7 | ASL | na | 10545 | 0.086 | 0.1422 | No |
| 8 | GOT2 | na | 11639 | 0.069 | 0.1264 | No |
| 9 | ABAT | na | 12337 | 0.059 | 0.1197 | No |
| 10 | GLUD1 | na | 12491 | 0.057 | 0.1283 | No |
| 11 | NIT2 | na | 13137 | 0.048 | 0.1208 | No |
| 12 | PPAT | na | 14277 | 0.035 | 0.0958 | No |
| 13 | GOT1 | na | 16062 | 0.017 | 0.0483 | No |
| 14 | ALDH4A1 | na | 16951 | 0.009 | 0.0248 | No |
| 15 | GLS | na | 18022 | -0.001 | -0.0059 | No |
| 16 | ADSL | na | 19439 | -0.015 | -0.0431 | No |
| 17 | GLUL | na | 20915 | -0.032 | -0.0782 | No |
| 18 | ASS1 | na | 21330 | -0.037 | -0.0816 | No |
| 19 | GLUD2 | na | 22397 | -0.051 | -0.1007 | No |
| 20 | GPT2 | na | 22719 | -0.055 | -0.0974 | No |
| 21 | GLS2 | na | 24209 | -0.079 | -0.1222 | No |
| 22 | ASNS | na | 24214 | -0.079 | -0.1042 | No |
| 23 | ALDH5A1 | na | 24768 | -0.089 | -0.0998 | No |
| 24 | GFPT1 | na | 25738 | -0.107 | -0.1032 | No |
| 25 | CAD | na | 27069 | -0.137 | -0.1103 | No |
| 26 | CPS1 | na | 28197 | -0.166 | -0.1048 | No |
| 27 | AGXT | na | 28253 | -0.168 | -0.0680 | No |
| 28 | ACY3 | na | 30672 | -0.249 | -0.0808 | No |
| 29 | GFPT2 | na | 30913 | -0.259 | -0.0285 | No |
| 30 | ASPA | na | 34315 | -0.606 | 0.0119 | No |
Table: GSEA details [plain text format]

  

Fig 2: KEGG\_ALANINE\_ASPARTATE\_AND\_GLUTAMATE\_METABOLISM      
 Blue-Pink O' Gram in the Space of the Analyzed GeneSet

  

Fig 3: KEGG\_ALANINE\_ASPARTATE\_AND\_GLUTAMATE\_METABOLISM: Random ES distribution      
 Gene set null distribution of ES for **KEGG\_ALANINE\_ASPARTATE\_AND\_GLUTAMATE\_METABOLISM**
